# Bibliographical

**Published:** 1890-10

**Authors:** 


### Bibliographical.

*The Student's Manual and Hand-Book for the Dental Laboratory, Second Edition, by L. P. Haskell, Prof. of Prosthetic Dentistry, Dental Department of the Northwestern University, Chicago, to which is appended Dr. E. H. Angle's System of Appliances for Correcting Irregularities.*

The first edition of this work having been exhausted some time ago, a second was called for, and it is gratifying that the call is so promptly answered. The work is a most excellent one, not only for the student, but for the dental practitioner as well. It is eminently a practical work; is the outcome of the experience and most faithful devotion to this line of dental practice, by one who has given it his entire attention for more than forty years.

The methods here given are all practical, and such as will secure in the hands of the trained manipulater the best results. The work is systematically arranged, and is made in every particular so plain and understandable that any one of ordinary intelligence on the subject can readily understand and utilize the methods set forth. The work is a reference book for the dental laboratory.

It does not, however, supersede other works that have been written on "Prosthetic Dentistry," but it occupies a place that has not been filled by any one hitherto. This work is the embodiment of Dr. Haskell's experience in Prosthetic Dentistry, and no dentist or student can in justice to himself, afford to be without this guide-book.

It is published by the Wilmington Dental Manufacturing Co., Philadelphia, and may be procured through any dental depot, or of Dr. Haskell.

